# Regorafenib treatment for advanced, refractory gastrointestinal stromal tumor: a report of the UK managed access program

**DOI:** 10.1186/2045-3329-4-17

**Published:** 2014-12-04

**Authors:** Attila Kollàr, Marco Maruzzo, Christina Messiou, Elisabeth Cartwright, Aisha Miah, Juan Martin-Liberal, Khin Thway, Ellen McGrath, Alison Dunlop, Komel Khabra, Beatrice Seddon, Palma Dileo, Mark Linch, Ian Judson, Charlotte Benson

**Affiliations:** Department of Medical Oncology, University Hospital Bern, Inselspital, 3010 Bern, Switzerland; Sarcoma Unit, Royal Marsden NHS Foundation Trust, Fulham Road, SW3 6JJ London, UK; University College London Hospitals NHS Foundation Trust, Euston Road, NW1 2BU London, UK; Research Data Management and Statistics Unit (RDSU), The Royal Marsden NHS Foundation Trust, London, UK

**Keywords:** Gastrointestinal stromal tumor, Regorafenib, Mutation, Choi

## Abstract

**Background:**

Tyrosine kinase inhibitors (TKI) have revolutionized the treatment of gastrointestinal

stromal tumors (GIST) although most patients develop resistance to first and second-line therapies.

Regorafenib, an oral multi-targeted TKI, has demonstrated benefit in previously treated GIST patients.

**Methods:**

We assessed safety and activity of regorafenib in patients treated within the Managed Access Program (MAP). All consecutive patients with advanced GIST who had progressed on or were intolerant to imatinib and sunitinib were recruited from the Royal Marsden and University College Hospitals. We retrospectively reviewed the data for response, toxicity, treatment duration and survival. Response was assessed by RECIST and Choi criteria. Toxicity was graded according to CTCAE v4.0 criteria.

**Results:**

20 patients were included in the MAP in the UK between 3/2013 and 9/2013. Median age was 68 (range 45–87), 65% of patients were male. Performance Status was 0–1 for 18 patients (90%), 2 for 2 patients (10%). The median treatment duration was 9.25 months (range 0.1-15.33). 18 patients were assessable for response and all patients attained a best response of at least stable disease. At a median follow-up of 12.6 months, there were 2 partial responses (11%) by RECIST and 7 partial responses (39%) according to Choi criteria. 7 patients remain on regorafenib. 3 patients discontinued treatment due to unacceptable adverse events; fistulation, myalgia and fatigue. 10 (50%) patients had grade 3 toxicities and 11 (55%) patients required a dose reduction. Median PFS was 9.4 months (95% Cl: 6.2-not calculable) and median OS was 12.2 months (95% Cl: 10.5-not calculable). Notably, prolonged stable disease was seen in 1 patient with exon 9 mutation and 1 patient with PDGFR D842V mutation.

**Conclusions:**

These data demonstrate encouraging activity and tolerability of regorafenib in routine clinical practice. The documented adverse events are in line with previous trial data.

## Background

Gastrointestinal stromal tumors (GIST) are the most common mesenchymal neoplasm in the gastrointestinal tract with a worldwide incidence of approximately 15 cases per million per year [1–3]. GIST are thought to be derived from the interstitial cells of Cajal that coordinate the peristaltic action of the gastrointestinal tract [4]. In localized disease, the primary treatment modality is surgery but despite complete resection, disease recurrence or metastasis occurs in more than 40% of patients [3]. In this advanced disease setting, cytotoxic chemotherapy has been largely ineffective [5, 6] however, molecular characterization of GIST has transformed the therapeutic landscape [7]. The majority of GIST have activating mutations in the genes for stem cell factor receptor (*KIT,* 85%) or platelet derived growth factor receptor α (*PDGFRA,* approximately 6-8%) and more rarely exclusive mutations in BRAF, NF1 and succinate dehydrogenase are found [2, 8–12]. Introduction of imatinib, a small molecule inhibitor of KIT and PDGFRα has had a major impact in this disease and is the standard 1^st^ line therapy [13]. Sunitinib, a multikinase inhibitor, is approved as second line therapy after disease progression during imatinib or for patients that are imatinib-intolerant [14].

Recently, regorafenib was introduced as a novel, oral multikinase inhibitor whose effects are achieved by targeting angiogenic (VEGFR1–3 and TEK), stromal (PDGFR and FGFR) and oncogenic (KIT, RET, RAF1, BRAF) receptor tyrosine kinases [15–18]. Preclinical data has shown that regorafenib has antitumor activity against human GIST xenografts [19].

Following a phase I trial, which led to the recommended dosing schedule of 160 mg OD 3 weeks on and 1 week off, George and colleagues published the results of the multi-centre phase II trial investigating the role of regorafenib in metastatic and/or unresectable GIST with progression on or intolerance to imatinib and prior failure of sunitinib. 12% (4/33) of the patients achieved a partial response (PR) per RECIST 1.1. and 66% (22/33) experienced stable disease (SD) for longer than 16 weeks. Median progression-free survival was 10.0 months [20, 21].

Based on these promising results an international randomized, double-blind, placebo-controlled phase III trial (GRID) was conducted. 199 patients with metastatic or unresectable GIST were randomized to receive 160 mg of regorafenib or placebo, after failure of imatinib and sunitinib. The median PFS was 4.8 months for regorafenib vs 0.9 months for placebo. Overall survival at the cutoff time was similar in both study arms, 22% events in the regorafenib and 26% in the placebo group, respectively. The median daily drug dose was 146.8 mg. The two most commonly reported drug-related adverse events were hand-foot-syndrome and hypertension [22].

Here we describe the safety and efficacy of regorafenib in patients enrolled in a managed access program (MAP), which was offered after the results of the GRID trial have been published and before the commercial launch. The MAP provided an opportunity to assess regorafenib in a cohort of advanced GIST patients in a routine clinical setting who had no other approved therapeutic options.

## Methods

### Study design and population

The inclusion criteria were in the main in accordance with the GRID trial [22]. In short, patients were eligible if they were at least 18 years of age with histologically confirmed metastatic and/or unresectable GIST, had progressed on or were intolerant of imatinib and/or sunitinib, had a performance status of 0–2 and had adequate haematological, liver and renal function. Relevant exclusion criteria included uncontrolled hypertension and any illness or medical condition that was unstable or could jeopardize the safety of the patient and his/her compliance in the program.

We reviewed the data of all the consecutive patients recruited into the MAP at the Royal Marsden and University College London Hospitals. Patients’ data were prospectively collected in the hospital electronic patient records systems. Local institutional ethical approvals were obtained.

### Treatment

All patients gave informed consent for treatment. Regorafenib was started at the full dose of 160 mg orally once daily for the first 3 weeks of each 4 week cycle. Initial dose reductions were permitted depending on PS and/or comorbidities according to the treating clinician’s discretion. Patients were treated continuously until disease progression or unacceptable toxicities. Dose reductions were implemented for moderate or severe toxicity. During the MAP period, regorafenib was provided by Bayer plc.

### Assessments

Tumor assessments were conducted with repeated computer tomography (CT) scan and or magnetic resonance imaging (MRI) at baseline and subsequently after every 2 cycles of treatment. Radiologic responses were assessed according to RECIST 1.1 criteria [23] and Choi criteria [24] by a single specialist Sarcoma Radiologist (C.M.).

Adverse events were monitored in all patients who received regorafenib and graded according to the Common Terminology Criteria for Adverse Events (CTCAE) version 4. All adverse events were recorded from the first intake of regorafenib until treatment discontinuation.

### Statistical analysis

Patient and disease characteristics were analysed using descriptive statistics. Overall survival (OS) was defined from date of starting treatment to date of death. Any surviving patients were censored at last follow up. Progression free survival (PFS) was defined from date of starting treatment to date of progression, assessed by RECIST 1.1., or death. Any progression free surviving patients were censored at last follow up. PFS and OS were estimated using Kaplan-Meier analysis. Median follow up was calculated using the inverse Kaplan Meier method and the median rates and 6 month rates are presented along with the 95% Confidence Intervals (CI).

## Results

### Patient characteristics

Between March and September 2013, 20 consecutive patients with a diagnosis of metastatic GIST were included in the MAP. Baseline characteristics are provided in Table 1. Median age was 68 years (range 45–87). 13 (65%) were male and 7 (35%) female. Most patients had an Eastern Cooperative Oncology Group (ECOG) PS of 0 or 1, with 2 patients (10%) having a PS of 2. All the patients had documented radiological progressive disease within three months prior to starting on regorafenib. GIST was of gastric origin in 8 (40%) patients. The remaining patients had GIST originating from small bowel, retroperitoneal, esophagogastric and one pelvic/rectal origin. Mutational analysis was available for 14 patients, with a majority of exon 11 mutations (60%); one patient had a mutation in the exon 9 (5%) and another one had a PDGFRA D842V mutation (5%). All the patients received prior treatment with imatinib and sunitinib. Median time on imatinib and sunitinib treatment was 37 months (range 2–87) and 11 months (range 2–46), respectively. Four patients (20%) received another line of treatment before regorafenib was initiated; 3 of them in a phase I setting and one patient received nilotinib. Median year of first diagnosis of GIST was 2006 (range 2000–2012). 5 patients received post-study treatment; four with imatinib alone or with a phase I investigational agent and one patient radiotherapy.

**Table 1:** **Patient characteristics**

Total	20
Age (median, range)	68 (45-87)
	n (%)
Sex	
Male	13 (65%)
Female	7 (35%)
ECOG performance status at baseline	
0-1	18 (90%)
2	2 (10%)
Site of primary	
Gastric	8 (40%)
Non-gastric	12 (60%)
Previous treatment lines	
2	16 (80%)
3	4 (20%)
Mutational analysis	
KIT exon 11 mutation	12 (60%)
KIT exon 9 mutation	1 (5%)
PDGFR D842V mutation	1 (5%)
Not avalilable	6 (30%)

### Dosing and toxicity

The median treatment duration was 9.25 months (range 0.1-15.33). 15 (75%) patients started at the full dose of 160 mg. Starting dose was lower in 5 patients due to PS, age and persisting toxicity from previous treatment; 3 patients started at 120 mg (15%) and 2 at 80 mg (10%) of regorafenib. 11 (55%) and 2 (10%) patients received dose modification during the treatment, with dose reduction or escalation, respectively. 3 patients (15%) discontinued treatment during the first month of treatment. One patient experienced an exacerbation of a known fistula, one suffered from severe pelvic pain and one from increasing fatigue. In all patients, drug-related adverse events of any grade were reported. The most common adverse events of any grade were fatigue in 16 (80%) patients, hand-foot-skin reaction in 11 (55%) patients and hypertension and diarrhea in half of the patients. Laboratory abnormalities of any grade were documented in 2 patients in terms of elevated alkaline phosphatase and hyperbilirubinemia. Grade 3 toxicities were documented in 10 patients (50%). 3 patients (15%) each experienced hand-foot-skin reaction and hypertension, respectively, 2 (10%) patients reported skin rash, 1 patient (5%) had fatigue, one patient (5%) had oral mucositis and in one patient a hyperbilirubinemia was reported. No grade 4 toxicities or toxic deaths were reported. Toxicities are summarized in Table 2.

**Table 2:** **Toxicity – number of patients/proportion out of all 20 patients**

Toxicity	All grades	Grade 3-4
Fatigue	16 (80%)	1 (5%)
Hand-foot-syndrome	11 (55%)	3 (15%)
Hypertension	10 (50%)	3 (15%)
Diarrhoea	10 (50%)	-
Oral mucositis	8 (40%)	1 (5%)
Hoarseness	8 (40%)	-
Constipation	7 (35%)	-
Rash, maculopapular	6 (30%)	2 (10%)
Anorexia	5 (25%)	-
Nausea	4 (20%)	-
Hepatobiliary toxicity	2(10%)	1(5%)

### Efficacy

**Figure 1:**
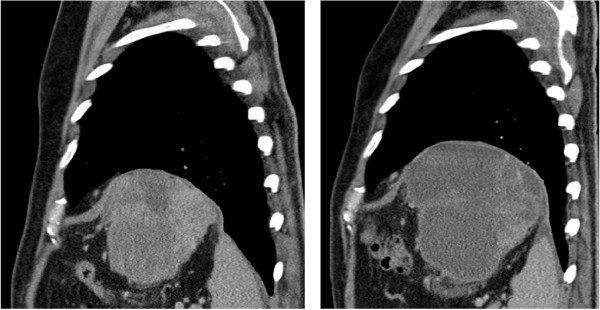
**Sagittal CT image of left upper quadrant mass.** Although the mass has increased in size, the drop in Hounsfield units from 39 to 16 suggests partial response by Choi criteria.

**Figure 2:**
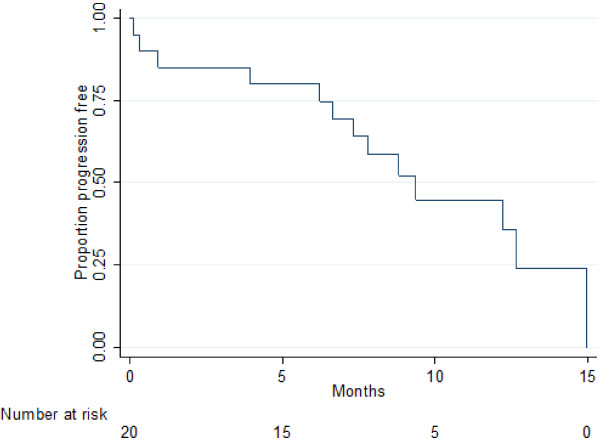
**Median PFS: 9.4 months (95% CI: 6.2 – not calculable).**

**Figure 3:**
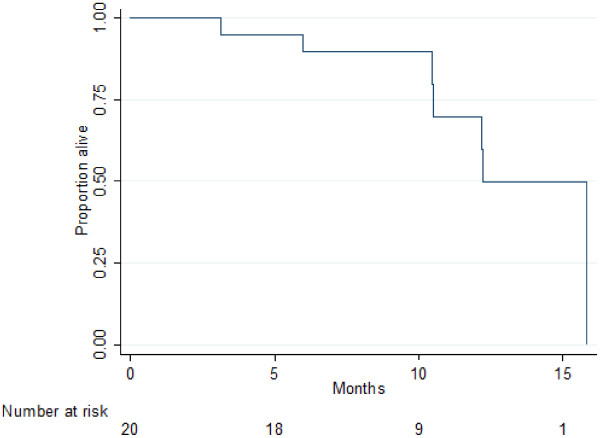
**Median OS: 12.2 months (95% CI: 10.5 – not calculable).**

After a median follow-up of 12.6 months, 18/20 patients were assessable for response according to RECIST and Choi criteria. Two patients were not assessable because of early discontinuation before radiological assessment. All assessable patients attained a best response of at least stable disease. Two partial responses (11%) were documented according to RECIST. In comparison, assessment by Choi criteria revealed a partial response in 7 patients (39%). An example of radiological response by Choi is illustrated in Figure 1. Notably, in the 4 patients that gained an improvement in PS, there was at least SD by both RECIST (2 PR, 2 SD) and Choi (3 PR, 1 SD) criteria. At the time of analysis (14.06.2014), 13 (65%) patients had discontinued regorafenib and 7 (35%) patients were still benefiting from regorafenib treatment. The median PFS was 9.4 months (95% Cl: 6.2-not calculable) and the 6-month PFS was 80.0% (95% CI 55.1-92.0) (Figure 2). The median OS was 12.2 months (95% Cl: 10.5-not calculable) and the 6-month OS rate was 89.7% (95% CI 64.8-97.3) (Figure 3).

## Discussion

Regorafenib is a multi-targeted tyrosine kinase inhibitor with evidence of non-cross resistant activity in patients with previously treated GIST. The phase III clinical trial of regorafenib in GIST demonstrated a statistically significant improved progression free survival in the third line setting for patients with GIST following failure of imatinib and sunitinib [22]. Its broad spectrum targeting pathways involved in oncogenesis have made this drug very appealing. In the MAP, patients with no other therapeutic options had the opportunity to benefit from regorafenib. We were able to assess efficacy and safety of regorafenib treatment in a routine clinical practice setting at two separate sarcoma centers in the UK.

Median treatment duration of regorafenib in the GRID trial was reported to be 22.9 weeks which is approximately 3 months less than in our cohort. According to the blinded central review and the investigator based assessment median PFS in the phase III trial was 4.9 months and 7.4 months, respectively, with a PFS at 6 months of 60%, also less than in our analysis. However these differences cannot be meaningfully compared due to our smaller patient numbers and less rigorous inclusion and stopping criteria. Interestingly a larger number of patients received more than 2 previous treatment lines in the GRID trial that could also account for such a difference. Complete radiological responses were not seen in either the GRID trial, or in the MAP. Twice as many partial responses were documented by RECIST criteria in our cohort in comparison with the trial setting, 11% and 4.5%, respectively. Additionally, we assessed response according to the Choi criteria taking into account the specific radiological changes of GIST during TKI therapy. As expected, the partial response rate according to Choi criteria was increased compared with RECIST 1.1. Shinagare and colleagues reported recently on the correlation of different assessment techniques with the outcome. Whereas PR was more frequent by Choi (90%) than RECIST 1.1, disease control rate was similar between the different tumor response criteria. Interestingly, the concordance of RECIST 1.1 evaluation with PFS and OS was more exact than with Choi [25]. Our data confirm that regorafenib has the potential to control disease over many months. There were not enough patients to make a fair comparison between the two assessment criteria. However, the correlation between PS improvement and response was higher when using response evaluation by Choi criteria.

Notably, we included one patient with an exon 9 and one with a PDGFRA D842V mutation. Both patients experienced prolonged stable disease for 8 and 12 months, respectively. A PR by Choi criteria was documented in the PDGFRA mutated case. In five patients with primary resistance to sunitinib, disease stabilization and even partial responses by Choi and RECIST criteria were documented on regorafenib treatment suggesting non-cross resistance of these agents. These findings support the strategy that is currently being tested of alternating sunitinib and regorafenib in an attempt to try and overcome acquired resistance mutations (NCT02164240).

All patients treated within the MAP experienced mild adverse events. The documented toxicities, particularly of grade 3, are consistent with previous published data. Fatigue, hoarseness, constipation and rash were more often reported in our cohort than in the GRID trial, but are similar to other published regorafenib studies [8, 20, 21, 26–28]. The need to reduce the standard treatment dose of 160 mg in more than half of the patients (55%) suggest that the optimal dose of regorafenib may be less than 160 mg. Patients that received 120 mg once daily rarely suffered with ≥ grade 3 toxicities. The median daily dose of regorafenib in the GRID trial was reported to be 146.8 mg in concordance with our observation. Notably, our experience supports the perception that the majority of adverse events are reversible and manageable with dose delays, dose reductions and intensified supportive care [29]. We performed clinical and laboratory assessment of adverse events every two weeks during the first 3 cycles. Specialist nursing input was available for patients receiving regorafenib, helping to raise patient awareness of expected side-effects and advise on toxicity management in a timely fashion. We feel that these intensive early assessments coupled with the nursing support plays a crucial part in successful management regorafenib therapy.

Importantly for a trial of a palliative therapy, the GRID study included quality of life outcomes, albeit only as exploratory endpoints. Poole and colleagues reported the QoL data from assessments of global health status and the physical functioning domain scores and demonstated no statistical differences between the regorafenib and placebo cohorts highlighting that regorafenib treatment toxicities do not have a negative impact on QoL [30]. In our study, formal QoL assessment was not performed but PS was rigorously documented. Four out of 20 patients (20%) had improved their general status consistent with the results seen in the GRID trial. This observation that regorafenib is a well-tolerated treatment that leads to symptomatic benefit in a sizeable proportion of patients in this study strengthens the role of regorafenib in routine clinical practice. Furthermore, the fact that patients with a PS of 2 were included in the MAP and that their adverse events were successfully managed makes regorafenib treatment feasible in a real world population. We feel that this toxicity management should include close observation and consideration of a reduced initial dose of regorafenib in patients who are PS 2 or who have suffered with previous TKI toxicity. According to the known phase III trials (CORRECT and GRID) there seems to be no worsening of regorafenib toxicity following treatment with other tyrosine kinase inhibitors like imatinib or sunitinib [22, 28].

## Conclusions

In conclusion, our data confirms the efficacy and safety data of the GRID trial supporting the use of regorafenib in a real world setting including a range of differing mutational subtypes. Additional data regarding the efficacy of regorafenib in relation to the mutational status is needed.

## Abbreviations

UK: United Kingdom
EMEA: European medicines agency
MAP: Managed access program
GIST: Gastrointestinal stromal tumor
TKI: Tyrosine kinase inhibitor
PDGFRA: Platelet-derived growth factor receptor α
CT: Computer tomography scan
MRI: Magnetic resonance imaging
CTCAE: Common terminology criteria for adverse events
RECIST: Response evaluation criteria in solid tumors
ECOG: Eastern cooperative oncology group
PS: Performance score
OS: Overall survival
PFS: Progression-free survival
CI: Confidence interval
PR: Partial response
SD: Stable disease
QoL: Quality of life.
